# Translational and real-world evidence of trastuzumab biosimilar CT-P6 plus pertuzumab in neoadjuvant HER2-positive early breast cancer

**DOI:** 10.1007/s10549-026-07895-8

**Published:** 2026-01-20

**Authors:** José Luis Alonso-Romero, Jerónimo Martínez-García, Raúl Carrillo-Vicente, Antonio Fernández Aramburo, Angélica Ferrando Díez, Pilar Sánchez Henarejos, Pilar de la Morena Barrio, Ana Puertes Boix, Mª Dolores Jiménez, Joaquín Peña Siles, José Antonio Parejo Maestre, Antonio de las Heras-Rubio, Paula Ruiz Carreño

**Affiliations:** 1 Department of Oncology, Hospital Clínico Universitario Virgen de la Arrixaca, Ctra. Madrid-Cartagena s/n. 30120. (HCUVA), Murcia, Spain; 2 Department of Oncology, Instituto Murciano de Investigación Biosanitaria Pascual Parrilla, Murcia, Spain; 3 Department of Oncology, HU Santa Lucía, Cartagena, Spain; 4 Department of Oncology, CHU Albacete, Albacete, Spain; 5 Department of Oncology, Institut Català d’ Oncologia (ICO), Badalona (Barcelona), Spain; 6 Department of Oncology, Hospital General Universitario Morales Meseguer, Murcia, Spain; 7 SCORE Lab, I3US Institute, University of Sevilla, Seville, Spain; 8 Medical Science Department, Scientific and Technical Area, Kern Pharma S.L, Barcelona, Spain

**Keywords:** HER2-positive early breast cancer, Neoadjuvant treatment, Routine clinical practice, Trastuzumab biosimilar, Trastuzumab CT-P6

## Abstract

**Background:**

Data on neoadjuvant treatment with trastuzumab biosimilars, particularly CT-P6, in combination with pertuzumab, are limited. This study evaluates the efficacy, tolerability, and immunogenicity of CT-P6 plus pertuzumab and chemotherapy, in routine clinical practice for HER2-positive early breast cancer, including translational biomarker analyses related to pathologic complete response (pCR).

**Methods:**

Prospective, multicenter, observational study in 102 patients with HER2-positive early breast cancer. Patients received hospital-preferred neoadjuvant regimens protocols, with (scheme 1 and 3) or without anthracyclines (scheme 2). The primary endpoint was pCR, defined as the absence of invasive tumor in both the breast and axillary lymph nodes (ypT0/ypTis and ypN0). Translational endpoints included soluble HER2, anti-trastuzumab CT-P6 antibodies, and exploratory response-related modeling approaches supported by machine learning techniques.

**Results:**

Among patients who underwent surgery, pCR (ypT0/ypTis and ypN0) was achieved in 57.43% of cases, with no significant differences between anthracycline-based and non-anthracycline-based regimens. Soluble HER2 and anti-trastuzumab CT-P6 antibodies were not significantly associated with pCR. Treatment was well-tolerated; the most relevant Grade 3–4 treatment-related adverse events were diarrhea (2.25%) and asthenia (0.50%). No immunogenicity or clinically relevant cardiotoxicity was observed.

**Conclusions:**

Trastuzumab CT-P6 combined with pertuzumab and chemotherapy can be used in neoadjuvant treatment for HER2-positive early breast cancer, showing pCR rates comparable to the reference trastuzumab and without evidence of immunogenicity. Exploratory analyses of soluble HER2 and anti-trastuzumab CT-P6 antibodies did not demonstrate a significant association with pCR, although this possibility cannot be excluded. Their assessment contributes to the translational understanding of biosimilar integration into curative regimens.

**Trial registration:**

The study has been registered in Clinicaltrials.gov (https://clinicaltrials.gov/study/NCT06907082).

**Supplementary Information:**

The online version contains supplementary material available at 10.1007/s10549-026-07895-8.

## Background

Breast cancer is the most common malignancy in women in developed countries and the leading cause of cancer-related death in women. In these settings, most patients are diagnosed at an early-stage [1]. Human epidermal growth factor receptor 2 (HER2)-positive breast cancer is associated with a poor prognosis and accounts for 13–15% of cases [2, 3].

Randomized clinical trials have found no difference in long-term outcomes when chemotherapy is given before or after surgery [4]. Neoadjuvant chemotherapy (NACT) has traditionally been used to improve surgical outcome in locally advanced tumors, but it also provides important prognostic information based on treatment response and is associated with higher rates of breast preservation [5, 6]. HER2-targeted therapies, in combination with chemotherapy, have been widely used as a neoadjuvant systemic therapy regimen before surgery with the aim of improving operability and pCR [7]. Achievement of a pathologic complete response (pCR) after NACT is associated with increased progression-free survival and overall survival in HER2-positive breast cancer [4–8].

According to international clinical practice guidelines, neoadjuvant systemic therapy is indicated for HER2-positive early breast cancer when tumors are ≥  2 cm, node-positive, or when downstaging is needed for breast conservation, but is not routinely recommended for small (≤ 1 cm), node-negative tumors, for which primary surgery is generally preferred [4, 9].

For tumors that overexpress or amplify HER2, neoadjuvant treatment with chemotherapy combined with dual anti-HER2 blockade is indicated standard of care. This approach was endorsed by the 2017 St. Gallen Expert Consensus Conference and is supported by NCCN and ESMO guidelines [4, 9, 10]. Trastuzumab and pertuzumab are recombinant humanized monoclonal antibodies that target different extracellular regions of the HER2 receptor, resulting in complementary and synergistic inhibition of HER2 signaling [4, 9–11].

To date, different clinical trials have shown that neoadjuvant treatment with chemotherapy combined with dual blockade of HER2-positive tumors using pertuzumab and trastuzumab provides a high rate of pCR, with an acceptable tolerance. Although a slight increase in left ventricular ejection fraction (LVEF) decline has been reported, rare events of clinical cardiac dysfunction have been reported [12–16]. However, a standard chemotherapy regimen to accompany dual anti-HER2 blockade has yet to be established.

The emergence of biosimilars in general, particularly trastuzumab biosimilars, is contributing to the economic sustainability of healthcare systems. Beyond demonstrating comparable clinical efficacy and safety to the originator molecule, the integration of biosimilars into curative settings requires robust translational evaluation, including analyses of immunogenicity and biomarkers, to ensure their reliability in combination regimens.

CT-P6 (trastuzumab-pkrb, Herzuma®, Celltrion, South Korea) is a trastuzumab biosimilar approved by the European Medicines Agency (EMA) for use in the same indications as the original trastuzumab product [17]. Preclinical development and clinical studies revealed no differences between CT-P6 and the reference trastuzumab [18].

Currently, the information available on the use of trastuzumab biosimilars in combination with pertuzumab in the neoadjuvant setting remain limited, particularly for CT-P6. To date, no prospective study has explored not only the real-world efficacy of trastuzumab CT-P6 in combination with pertuzumab, but also its immunogenicity profile and potential translational biomarkers of response in the neoadjuvant setting.

Therefore, we designed a prospective study to analyze the use of trastuzumab CT-P6 in combination with pertuzumab and chemotherapy in routine clinical practice (RCP). The objectives were to assess efficacy, tolerability, and immunogenicity data to corroborate the safe use of trastuzumab CT-P6 in the neoadjuvant setting and to explore clinical and biomarker factors potentially associated with pCR with this combination.

## Methods

### Study design and participants

This was a prospective, observational (non-interventional), open-label, multicenter study conducted in 5 Spanish hospitals with experience in breast cancer treatment. Patients were consecutively enrolled between December 2020 and May 2024. All participants had HER2-positive early breast cancer amenable to neoadjuvant treatment with chemotherapy and dual anti-HER2 blockade. The study was registered in Clinicaltrials.gov (https://clinicaltrials.gov/study/NCT06907082).

Eligible patients were women aged 18 years or older, with histologically confirmed early-stage HER2-positive invasive breast cancer, defined as clinical anatomic stage II or III according to the American Joint Committee on Cancer (AJCC) staging system, who were candidates for neoadjuvant treatment with chemotherapy and dual anti-HER2 blockade with pertuzumab and trastuzumab CT-P6 according to ESMO guideline 2019 [9]. Patients with small ( ≤ 1 cm), node-negative tumors (T1a-bN0), for whom neoadjuvant therapy is not routinely recommended, were not included. Exclusion criteria included metastatic breast cancer, known hypersensitivity to trastuzumab or pertuzumab, current treatment with an investigational agent, diagnosis of any other neoplastic pathology of prognostic relevance within the previous two years except cervical or breast carcinoma in situ and basal cell or squamous cell carcinoma of the skin, non-neoplastic pathology with a life expectancy of less than one year, and pregnancy or lactation.

The study was conducted in full compliance with the principles of the Declaration of Helsinki and the Good Pharmacoepidemiology Practices (GPP), as well as with all Spanish legislation applicable to observational studies (Order SAS/3470/2009). The study was evaluated and approved by the Ethics Committee (2020–3-2-HCUVA) of the Hospital Clínico Universitario Virgen de la Arrixaca, Murcia, Spain. All patients provided written informed consent.

### Procedures and outcomes

HER2 status was determined on pretreatment tumor samples in accordance with current ASCO/CAP guidelines. Immunohistochemistry (IHC) was performed as the initial diagnostic test [19]. Tumors with an IHC score of 3+  were considered HER2-positive. Cases with equivocal HER2 expression (IHC 2+ ) underwent confirmatory in situ hybridization (ISH/FISH), and HER2 positivity was defined by evidence of gene amplification according to ASCO/CAP criteria.

Peripheral blood samples for translational analyses were collected prior to initiation of neoadjuvant therapy. Blood was drawn into EDTA-containing tubes, centrifuged within 15 min of collection, and plasma aliquots were stored at  −  80 °C until analysis.

Soluble HER2 (sHER2) plasma levels were measured using a commercially available enzyme-linked immunosorbent assay (ELISA) (Protein Tech, Cat. KE00053, no RRID available) according to the manufacturer’s instructions. Results were expressed in ng/mL, and assay sensitivity and limits of quantification were defined according to the manufacturer’s specifications.

Anti-trastuzumab CT-P6 antibodies were assessed using a validated immunoassay platform (Krishgen Biosystems, Cat. KBI2017, KRIBIOLISA™ Anti-Trastuzumab/Herceptin ELISA, no RRID available). Samples were classified as positive or negative based on predefined assay cutoffs. All biomarker analyses were performed in a centralized laboratory.

Patients were treated in accordance with the preferred hospital protocol, primarily regarding the use or non-use of anthracyclines. Only three treatment schemes were allowed:

1. *Scheme 1*: Adriamycin 60 mg/m^2^ (or epirubicin 90 mg/m^2^)  +  cyclophosphamide 600 mg/m^2^ × 4 cycles, every 15 or 21 days (according to the usual clinical protocol of the service), followed by paclitaxel 80 mg/m^2^ weekly × 12 weeks (or docetaxel 100 mg/m^2^ every 3 weeks × 4), pertuzumab 840 mg in cycle 1 and 420 mg in cycles 2 – 4 (cycles every 21 days) + trastuzumab CT-P6 8 mg/kg in cycle 1 and 6 mg/kg in cycles 2–4 (cycles every 21 days).
2. *Scheme 2*: Docetaxel 75 mg/m^2^ every 3 weeks × 6 cycles  +  carboplatin 5 or 6 AUC every 3 weeks × 6 cycles  +  pertuzumab 840 mg in cycle 1 and 420 mg in cycles 2–6 (cycles every 21 days) + trastuzumab CT-P6 8 mg/kg in cycle 1 and 6 mg/kg in cycles 2–6 (cycles every 21 days).
3. *Scheme 3*: paclitaxel 100 mg/m^2^ weekly × 8 weeks  +  pertuzumab 840 mg in cycle 1 and 420 mg in cycles 2–3 (cycles every 21 days)  +  trastuzumab CT-P6 8 mg/kg in cycle 1 and 6 mg/kg in cycles 2–3 (cycles every 21 days) (both administered together with doses 1, 4 and 7 of paclitaxel), followed by epirubicin 90 mg/m^2^ every 3 weeks  +  pertuzumab and trastuzumab CT-P6 IV every 3 weeks, with the same doses  ×  4 cycles.

The primary endpoint was the pCR in breast tissue and axilla in patients with HER2-positive early breast cancer after neoadjuvant treatment. pCR was defined as the absence of infiltrating tumor cells in the breast tumor (ypT0/is) and any tumor cells in the axilla (ypN0).

Demographic, clinical, tumor, efficacy and toxicity data were collected. In addition to standard clinical outcomes, a translational substudy was integrated to assess soluble HER2 plasma levels, anti-trastuzumab CT-P6 antibodies, and exploratory prediction models using machine learning techniques. Three blood samples were additionally taken (immunogenicity and Her2 soluble protein): sample 1 (M1) or baseline sample obtained prior to the start of oncological treatment with dual anti-HER2 blockade, sample 2 (M2) obtained in cycle 4 of NACT with dual anti-HER2 blockade, and sample 3 (M3) obtained 10 days prior to surgery or at the post-surgical visit. The translational study added in this project was conducted concurrently with routine blood sampling, requiring no additional venipunctures.

### Statistical analysis

A sample size of 96 was planned considering a pCR rate of 52.8%, a precision of 10% in a bilateral analysis, an alpha risk of 0.05, and a power of 0.8 (estimating a 10% loss rate, a total sample size of 106 patients should be recruited).

A descriptive analysis was conducted in which quantitative variables were summarized using measures of central tendency and dispersion, including the mean, standard deviation, median, minimum, and maximum values. Categorical variables were described using absolute frequencies and corresponding percentages. For the purpose of analysis, treatment schemes 1 and 3 were grouped together, resulting in two comparison groups: Scheme 1 + 3 and Scheme 2, corresponding to anthracycline-based versus non-anthracycline-based regimens, respectively.

For the inferential analysis, parametric tests were applied to continuous variables, while nonparametric tests were employed for ordinal, categorical, or non-normally distributed variables. All hypothesis testing was conducted using two-tailed tests with a significance level set at 0.05. For variables that did not meet the assumptions of normality, the Mann–Whitney U test was used for unpaired data, and the Wilcoxon signed-rank test was applied for paired data. The comparison of categorical variables was performed using the Chi-square test or Fisher’s exact test, as appropriate.

The findings from the exploratory response prediction analysis were validated through the application of machine learning algorithms, including decision trees, random forest, and clustering techniques. The analysis was conducted using Python, along with relevant scientific libraries such as NumPy and *SciPy*.

## Results

### Patients and treatment

**Fig. 1:**
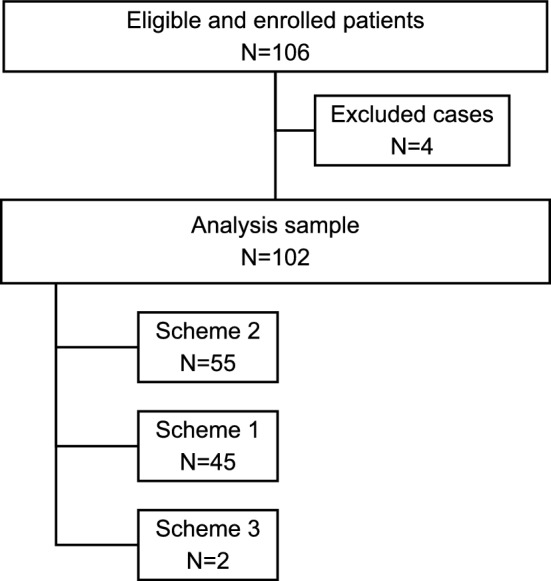
Flow diagram summarizing the research design

A total of 106 patients were included in the study. Four patients were excluded from the study (three withdrew their consent, and one received a different subcutaneous trastuzumab than the study treatment). Therefore, the final number of evaluable patients was 102. Patient inclusion, exclusion, and analysis are summarized in Fig. 1.

Patient characteristics at baseline are shown in Table 1. Since only 2 patients from a single hospital were included in Scheme 3, and as it was a scheme with anthracyclines and taxanes that was highly similar to Scheme 1, it was decided to merge Schemes 1 and 3 for analysis. However, the data were also analyzed independently and can be found in Supplementary Table S1. The median age of the patients was 52 years, ranging from 30 to 78 years old. Of the 102 patients receiving NACT, 55 (53.92%) received Scheme 2, and 47 (46.08%) received Scheme 1 + 3.

**Table 1:** Patient demographics at baseline

	All patients *n* = 102	NACT Scheme 2 *n* = 55	NACT Scheme 1 + 3 *n* = 47
Age (years), median (range)	52.00 (30–78)	56.00 (36–74)*	45.00 (30–78)*
0 to < 40	20 (19.61%)	6 (10.91%)	14 (29.79%)
40 to < 65	64 (62.74%)	37 (67.27%)	27 (57.45%)
≥ 65	18 (17.65%)	12 (21.82%)	6 (12.76%)
Menopausal state			
Pre-menopause	46 (45.10%)	17 (30.91%)*	29 (61.70%)*
Post-menopause	56 (54.90%)	38 (69.09%)*	18 (38.30%)*
ER			
Positive	58 (56.86%)	33 (60.00%)	25 (53.19%)
Negative	44 (43.14%)	22 (40.00%)	22 (46.81%)
PR			
Positive	37 (36.27%)	23 (41.82%)	14 (29.79%)
Negative	65 (63.73%)	32 (58.18%)	33 (70.21%)
ER and/or PR			
Positive	59 (57.84%)	33 (60.00%)	26 (55.32%)
Negative	43 (42.16%)	22 (40.00%)	21 (44.68%)
Ki67			
Ki67 < 20	20 (19.61%)	11 (20.00%)	9 (19.15%)
Ki67 ≥ 20	82 (80.39%)	44 (80.00%)	38 (80.85%)
HER2			
IHC 2+ (FISH amplified)	20 (19.61%)	11 (20.00%)	9 (19.15%)
IHC 3+	82 (80.39%)	44 (80.00%)	38 (80.85%)
Tumor distribution			
Unique	68 (66.66%)	36 (65.44%)	32 (68.08%)
Multifocal	19 (18.63%)	9 (16.36%)	10 (21.28%)
Multicentric	14 (13.73%)	9 (16.36%)	5 (10.64%)
Unknown	1 (0.98%)	1 (1.82%)	0 (0.00%)
Number of foci			
1	67 (66.34%)	37 (67.26%)	30 (65.21%)
2	15 (14.85%)	7 (12.73%)	8 (17.39%)
3	6 (5.94%)	2 (3.64%)	4 (8.70%)
> 4	8 (7.84%)	7 (12.73%)	1 (2.17%)
Unknown	5 (4.95%)	2 (3.64%)	3 (6.52%)
Size of biggest tumor (mm), median (range)	30.50 (10–110)	35.00 (11–90)	30.00 (10–110)
0 to < 20	14 (13.72%)	5 (9.09%)	9 (19.15%)
20 to < 50	71 (69.61%)	39 (70.91%)	32 (68.08%)
≥ 50	17 (16.67%)	11 (20.00%)	6 (12.77%)
Histologic grade			
G1	9 (8.82%)	3 (5.45%)	6 (12.77%)
G2	44 (43.14%)	21 (38.18%)	23 (48.94%)
G3	35 (34.31%)	29 (52.73%)	6 (12.77%)
GX	14 (13.73%)	2 (3.64%)	12 (25.53%)
Histological subtype			
Ductal NOS	94 (92.16%)	53 (96.36%)	41 (87.23%)
Other subtypes^a^	8 (7.84%)	2 (3.64%)	6 (12.7%)
cN			
0	50 (49.02%)	22 (40.00%)	28 (59.57%)
1	26 (25.49%)	16 (29.09%)	10 (21.28%)
2	16 (15.69%)	10 (18.18%)	6 (12.77%)
3	7 (6.86%)	6 (10.91%)	1 (2.13%)
x	3 (2.94%)	1 (1.82%)	2 (4.25%)
cT			
1	13 (12.74%)	7 (12.73%)	6 (12.76%)
2	66 (64.71%)	35 (63.63%)	31 (65.96%)
3	19 (18.63%)	10 (18.18%)	9 (19.15%)
4	2 (1.96%)	1 (1.82%)	1 (2.13%)
x	2 (1.96%)	2 (3.64%)	0 (0.00%)
Time to surgery (days), median (range)	68.50 (28–147)	56.00 (28–140)	84.00 (47–147)
Type of surgery^b^—breast			
Conservative surgery	63 (62.38%)	34 (61.82%)	29 (63.04%)
Mastectomy	38 (37.62%)	21 (38.18%)	17 (36.96%)
Type of surgery^b^ –—axilla			
SLNB	62 (62.63%)	26 (49.06%)	36 (78.26%)
Lymphadenectomy	37 (37.37%)	27 (50.94%)	10 (21.74%)
Number of cycles of CT-P6 + pertuzumab, median (range)	–	6.0 (4–6)	4.0 (3–4)

Data are number (%) unless otherwise specified. ^a^ Other histological subtypes include medullar, lobular, apocrine, and invasive carcinoma with micropapillary ductal and papillary type areas. ^b^ Of the 102 patients, 1 was not operated due to COVID-19; therefore, 101 patients underwent breast surgery and 99 underwent axillary surgery. * Indicates statistically significant difference between Scheme 2 and Scheme 1 + 3 (*p * < 0.05)

*cN* clinical node stage, *cT* clinical tumor stage, *ER* estrogen receptor, *FISH* fluorescence in situ hybridization, *G1* well-differentiated, *G2* moderately differentiated, *G3* poorly differentiated, *GX* not classified, *HER2* human epidermal growth factor receptor 2, *NACT* neoadjuvant chemotherapy, *NOS* no other specifications, *PR* progesterone receptor, *Scheme 2* with anthracyclines, *Scheme 1 + 3* without anthracyclines, *SLNB* sentinel lymph node biopsy

The comparison of the study population by Scheme found a significant difference in age (*p* = 0.001044), and in the menopausal state (*p* = 0.002655). Patients in Scheme 1 + 3 were younger, and there were more pre-menopausal patients (see Table 1).

The difference in the number of cycles of CT-P6 plus pertuzumab between Scheme 2 and Scheme 1 + 3 reflects the differences in chemotherapy backbone and treatment duration according to institutional protocols, with longer neoadjuvant exposure in anthracycline-containing regimens.

### Efficacy

Table 2 shows pCR results obtained after NACT with dual anti-HER2 blockade. Of the 102 patients included in the study, 101 underwent surgery. One patient, despite completing all other study procedures, was not operated due to a COVID-19 infection and was therefore excluded from the pathologic complete response evaluation.

**Table 2:** Pathologic complete response (pCR) after surgery

	All patients *n* = 101^a^	NACT Scheme 2 *n* = 55	NACT Scheme 1 + 3 *n* = 46^a^
pCR—Global			
Breast	61 (60.40%)	29 (52.73%)	32 (69.57%)
Axilla	81 (80.20%)	41 (74.55%)	40 (86.96%)
Breast and axilla	58 (57.43%)	29 (52.73%)	29 (63.04%)
pCR—HR negative			
Breast	35 (83.33%)	17 (77.27%)	18 (90.00%)
Axilla	37 (88.10%)	19 (86.36%)	18 (90.00%)
Breast and axilla	33 (78.57%)	17 (77.27%)	16 (80.00%)
pCR—HR positive			
Breast	26 (44.83%)	12 (37.50%)	14 (53.85%)
Axilla	44 (75.86%)	22 (68.75%)	22 (84.62%)
Breast and axilla	25 (43.10%)	12 (37.50%)	13 (50.00%)
ypT			
ypT0	55 (54.46%)	26 (47.27%)	29 (63.05%)
ypT1	32 (31.68%)	19 (34.55%)	13 (28.26%)
ypT2	8 (7.92%)	7 (12.73%)	1 (2.17%)
ypTis	6 (5.94%)	3 (5.45%)	3 (6.52%)
ypN			
0	81 (80.20%)	41 (74.55%)	40 (86.96%)
1	16 (15.84%)	10 (18.18%)	6 (13.04%)
2	2 (1.98%)	2 (3.64%)	0 (0.00%)
3	2 (1.98%)	2 (3.64%)	0 (0.00%)

Data are number (%) unless otherwise specified. ^a^ Of the 102 patients, 1 was not operated due to COVID-19, and therefore was not included in the pathologic complete response evaluation after surgery

*HR* hormone receptor, *NACT* neoadjuvant chemotherapy, *pCR* pathologic complete response, *ypN* lymph nodes in axilla, *Scheme 2* with anthracyclines, *Scheme 1 + 3* without anthracyclines, *ypT* residual tumor in breast tissue

The percentage of patients achieving pCR in both breast and axilla (ypT0 +  ypTis and ypN0) was 57.43% (58/101) overall, 52.73% (29/55) with Scheme 2, and 63.04% (29/46) with Scheme 1 + 3. When analyzed separately, pCR was achieved in the breast in 60.40% (61/101) of patients and in the axilla in 80.20% (81/101). Axillary response was assessed pathologically in patients who underwent axillary surgery (sentinel lymph node biopsy and/or axillary lymph node dissection), including those with clinically node-negative disease at baseline; ypN0 reflected absence of residual tumor or pathological treatment effect in resected lymph nodes.

The comparative analysis of Schemes 2 and 1  +  3 revealed no significant differences in pCR, regardless of whether the results were examined in the breast (*p*  =  0.149643), in the axilla (*p* = 0.204635), or in both the breast and axilla (*p*  =  0.417684).

Tumor size showed a significant association with pCR. In Scheme 1 + 3, larger tumor size was negatively associated with pCR in the breast and in the combined breast and axilla analysis (correlation − 0.300048, *p*  =  0.0053533 and  −  0.350816, *p*  =  0.022734, respectively). In Scheme 2, tumor size was also significantly associated with pCR in the breast and in the combined breast and axilla analysis (correlation − 0.449599, *p* = 0.01638 for both).

### Translational analysis

Translational endpoints included soluble HER2 and anti-trastuzumab CT-P6 antibody plasma levels, as well as exploratory prediction analyses validated with machine learning algorithms (due to the substantial size of the validation study employing machine learning techniques, the results will be incorporated into a subsequent publication).

Low-level anti-trastuzumab CT-P6 antibody signals were detected in a small proportion of samples across timepoints; however, no clinically meaningful differences were observed between treatment schemes, and no association with pCR was identified.

**Fig. 2:**
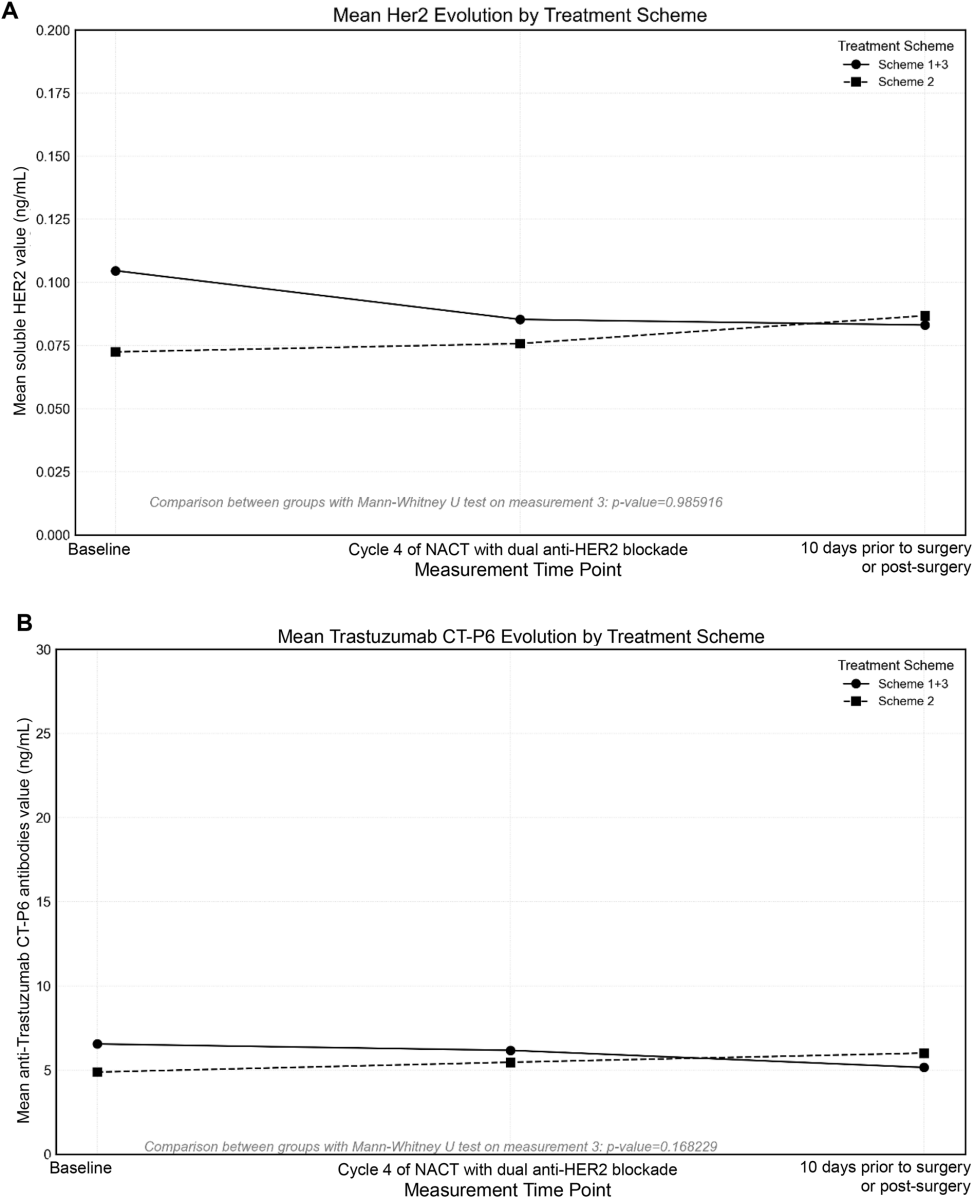
Evolution by TreatCment Scheme 1 + 3 and 2. **A** for soluble HER2. **B** for anti-Trastuzumab CT-P6 antibodies

Biomarkers were obtained in 97.06% (99/102) of patients at M1, 94.12% (96/102) of patients at M2, and 85% (90/102) of patients at M3. As shown in Table 3, the plasma concentrations of soluble HER2 and anti-trastuzumab CT-P6 antibodies are reported globally and by Scheme. Figure 1 illustrates the evolution of the mean plasma levels of soluble HER2 (Fig. 2A) and anti-Trastuzumab CT-P6 antibodies (Fig. 2B) by treatment Scheme. Soluble HER2 plasma concentration slightly increased in Scheme 2, while decreased in Scheme 1  +  3, but there were no significant differences between both Schemes. Also, anti-trastuzumab CT-P6 antibody plasma levels slightly increased in Scheme 2 and decreased in Scheme 1 + 3, and no significant differences were found between both Schemes.

**Table 3:** Soluble HER2 and anti-Trastuzumab CT-P6 antibodies plasma concentrations

	All patients *n* = 102	NACT Scheme 2 *n* = 55	NACT Scheme 1 + 3 *n* = 47
Soluble HER2 (ng/mL), median (range)			
M1	0.06 (0.00–0.81)	0.05 (0.00–0.39)	0.09 (0.00–0.81)
0.0 to < 0.01	5 (7.14%)	2 (7.69%)	3 (6.98%)
0.01 to < 1.0	65 (92.86%)	24 (92.31%)	40 (93.02%)
M2	0.05 (0.00–0.27)	0.04 (0.00–0.24)	0.06 (0.00–0.27)
0.0 to < 0.01	7 (9.86%)	3 (10.71%)	4 (9.30%)
0.01 to < 1.0	64 (90.14%)	25 (89.29%)	39 (90.70%)
M3	0.06 (0.00–0.41)	0.05 (0.00–0.41)	0.06 (0.00–0.36)
0.0 to < 0.01	6 (8.45%)	1 (3.57%)	5 (11.63%)
0.01 to < 1.0	65 (91.55%)	27 (96.43%)	38 (88.37%)
Anti-Trastuzumab CT-P6 antibodies (ng/mL), median (range)			
M1	5.38 (0.00–26.92)	5.38 (0.00–8.52)	5.00 (2.22–26.92)
0.0 to < 0.01	1 (1.43%)	1 (3.85%)	0 (0.00%)
0.01 to < 30.0	69 (98.57%)	25 (96.15%)	43 (100.00%)
M2	5.38 (0.00–20.00)	5.10 (0.00–20.00)	5.42 (2.59–17.31)
0.0 to < 0.01	1 (1.41%)	1 (3.57%)	0 (0.00%)
0.01 to < 30.0	70 (98.59%)	27 (96.43%)	43 (100.00%)
M3	5.00 (0.00–15.00)	5.80 (2.59–15.00)	4.62 (0.00–11.85)
0.0 to < 0.01	1 (1.41%)	0 (0.00%)	1 (2.33%)
0.01 to < 30.0	70 (98.59%)	28 (100.00%)	42 (97.67%)

Data are number (%) unless otherwise specified

*HER2* human epidermal growth factor receptor 2, *M1* measurement corresponding to the baseline sample obtained prior to the start of oncological treatment with dual anti-HER2 blockade, *M2* measurement corresponding to the sample obtained in cycle 4 of NACT with dual anti-HER2 blockade, *M3* measurement corresponding to the sample obtained 10 days prior to surgery or at the post-surgical visit, *NACT* neoadjuvant chemotherapy, *Scheme 2* with anthracyclines, *Scheme 1 + 3* without anthracyclines

No significant correlations were found between pCR and soluble HER2 (phi  =  0.334052), or with anti-trastuzumab CT-P6 antibodies (phi  =  0.331133).

### Safety

Globally, the most relevant treatment-related adverse events (AEs) in Scheme 2 were diarrhea (21.75%), mucositis (19.00%), nausea (17.88%), and asthenia (17.62%), while in Scheme 1  +  3 were asthenia (7.25%) and mucositis (5.25%) (see Table 4).

**Table 4:** More relevant treatment-related adverse events per cycle

	NACT Scheme 2 *n* = 55				NACT Scheme 1 + 3 *n* = 47			
	Global	Grade 1–2	Grade 3–4	Required admission	Global	Grade 1–2	Grade 3–4	Required admission
Diarrhea	21.75%*	19.38%*	2.25%*	0.88%*	4.00%*	4.00%*	0.00%*	0.00%*
Mucositis	19.00%*	18.62%*	0.00%	0.00%	5.25%*	5.25%*	0.00%	0.00%
Nausea	17.88%*	17.12%*	0.62%	0.25%	4.88%*	4.88%*	0.00%	0.00%
Asthenia	17.62%*	17.38%*	0.12%	0.25%	7.25%*	6.75%*	0.50%	0.00%
GI toxicity	10.88%*	10.62%*	0.12%	0.25%	2.62%*	2.38%*	0.00%	0.00%
Anemia	7.75%*	7.00%*	0.62%	0.00%	0.50%*	0.50%*	0.00%	0.00%
Neuropathic toxicity	5.58%*	5.75%*	0.00%	0.00%	3.12%*	3.12%*	0.00%	0.00%
Neutropenia	4.00%*	2.50%*	1.50%	0.12%	1.25%*	0.38%*	0.88%	0.00%
Neutropenic fever	0.00%	0.00%	0.25%	0.38%	0.00%	0.25%	0.12%	0.12%
LVEF alteration	0.00%	0.00%	0.00%	0.00%	0.00%	0.00%	0.00%	0.00%

Data represent the mean percentage of patients per treatment cycle. * Indicates statistically significant difference between Scheme 2 and Scheme 1 + 3 (*p*  < 0.05)

*GI* gastrointestinal, *LVEF* left ventricular ejection fraction, *NACT* neoadjuvant chemotherapy, *Scheme 2* with anthracyclines, *Scheme 1 + 3* without anthracyclines

The most relevant Grade 3–4 treatment-related AEs were diarrhea (2.25%) in Scheme 2 and asthenia (0.50%) in Scheme 1  +  3. The most relevant treatment-related AEs requiring hospital admission were diarrhea (0.88%) in Scheme 2 and neutropenic fever (0.12%) in Scheme 1  +  3.

The percentage of patients requiring a dose reduction was 32.73% in Scheme 2, and 22.45% in Scheme 1  +  3. The mean percentage of cycles with dose reduction was 40.09% for Scheme 2, and 30.13% for Scheme 1  +  3.

There were no deaths or AEs leading to death.

The comparison of treatment-related AEs rates between Scheme 2 and Scheme 1 + 3 revealed that Scheme 2 had a significantly higher proportion of AEs. Significant differences (*p*  < 0.05) were observed between the two schemes in nearly all parameters overall and for Grades 1–2, while for Grades 3–4, significance was found only for diarrhea.

Regarding cardiac safety, no significant issues were identified, and LVEF remained unaffected.

## Discussion

The primary objective of this study was to evaluate the efficacy, safety and immunogenicity of the trastuzumab CT-P6 in combination with pertuzumab and chemotherapy as neoadjuvant treatment in patients with HER2-positive early breast cancer in RCP according to pCR in breast and axilla. The results confirm that this regimen is effective, with 57% of all patients achieving full pCR both in breast and axilla, increasing to 63% of patients when receiving Scheme 1  +  3. Notably, when evaluated separately, while 60% of all patients achieved pCR in breast, a higher percentage of patients (80%) achieved pCR in axilla, consistent with known patterns of differential tumor response [20]. The high rate of axillary pCR observed in this cohort should be interpreted in the context of pathological assessment of resected lymph nodes following neoadjuvant therapy, including sentinel lymph node biopsy and/or axillary lymph node dissection, in both clinically node-positive and node-negative patients at baseline.

Despite the absence of a direct comparison with the original trastuzumab in this study, the results align with those of other published works using the original trastuzumab. The pCR results are similar although slightly lower than those found in the studies by Bernat-Peguera and Bae [21, 22]. These studies compared the pCR rate in HER2-positive early breast cancer patients treated with either trastuzumab CT-P6 or reference trastuzumab, and found that patients treated with CT-P6 achieved pCR rates of 65% and 74.4%, respectively.

This study is, to our knowledge, the first to prospectively evaluate immunogenicity and biomarker dynamics of a trastuzumab biosimilar in combination with pertuzumab in the neoadjuvant setting.

It is important to highlight the absence of immunogenicity measured in the context of this trastuzumab biosimilar. This study is the first to evaluate trastuzumab CT-P6 immunogenicity when combined with pertuzumab in a neoadjuvant setting. No clinically meaningful anti-trastuzumab CT-P6 antibody responses were detected in any patient during treatment, which corroborates the findings of previous studies that reported low immunogenic potential for trastuzumab CT-P6 [23, 24]. These findings reinforce the safety and reliability of this biosimilar, even when co-administered with another monoclonal antibody. The absence of clinically meaningful anti-trastuzumab CT-P6 antibody responses, even in the context of dual monoclonal antibody blockade, provides important translational reassurance of biosimilar safety at the immune interface.

Despite the disparities in age and menopausal status identified by Scheme, while Scheme 1  +  3 exhibited a preponderance of younger patients and a higher proportion of premenopausal subjects, these observations had no impact on the outcomes concerning efficacy, as no significant differences in pCR were observed between the two Schemes.

The results of the correlation analysis indicated a positive correlation between the extent of the tumor and the degree of response, with smaller tumors exhibiting higher levels of pCR. These findings are clinically reasonable and consistent with the observed responses.

The comparison of Scheme 2 and Scheme 1  +  3 with respect to soluble HER2 plasma concentrations and anti-trastuzumab CT-P6 antibody plasma levels revealed no significant differences. The slight increase in anti-trastuzumab CT-P6 antibody plasma levels observed in Scheme 2 could be attributed to the number of trastuzumab CT-P6  +  pertuzumab cycles administered to patients in this Scheme (median of 6 cycles), compared to Scheme 1  +  3 (median of 4 cycles). The stability of the anti-trastuzumab CT-P6 antibody levels, fluctuating within the range of 5 to 6, highlights the minimal immunogenic potential of trastuzumab CT-P6 and validates its safety profile.

The exploratory analyses of soluble HER2 and anti-trastuzumab CT-P6 antibody levels, although not significantly associated with pCR in this cohort, provide a framework for future translational studies of biosimilars aimed at further evaluating the clinical relevance of these biomarkers.

Regarding safety, the adverse events profile found in the present study was low and similar in patients treated with Scheme 1  +  3 and with Scheme 2, although the incidence of AEs and SAEs was a little higher in patients treated with Scheme 2. There were only 2 participants included in Scheme 3, so that no conclusions can be drawn for this group alone. The combination of trastuzumab CT-P6 and pertuzumab was generally well-tolerated, whether or not it was administered sequentially or concomitantly with chemotherapy in any of the three Schemes, which confirms the results obtained in a similar study with trastuzumab and pertuzumab also given sequentially or concomitantly with chemotherapy in three different arms and in the corresponding long-term study [12, 13].

Of particular relevance is the absence of cardiotoxicity in the present study. In a previous study with the original trastuzumab [25], heart failure episodes were not observed; however, a decrease in LVEF was documented in 3 of 70 patients. In the present study, no patient exhibited any heart failure or LVEF alteration in response to any of the NACT Schemes received. This finding serves to reinforce the cardiac safety of trastuzumab CT-P6, even when it is utilized in conjunction with pertuzumab and potentially cardiotoxic chemotherapies.

Exploratory response association analysis did not yield statistically significant results. Soluble HER2 was not significantly associated with pCR, although its potential clinical relevance cannot be excluded and therefore warrants further investigation. Exploratory analysis aimed at assessing response-related patterns, supported by machine learning algorithms, may assist in the future identification of patients more likely to benefit from treatment. However, due to the substantial size of the validation study employing machine learning techniques, detailed modeling results will be incorporated into a subsequent publication. While exploratory and underpowered, the incorporation of machine learning approaches illustrates the feasibility of integrating computational oncology tools into translational biosimilar research, paving the way for future precision-based modeling approaches in HER2-positive breast cancer.

A potential limitation of the study is the observational design, which precludes causal inferences. However, it should also be noted that this is a study conducted in RCP, outside the clinical trial setting, and therefore the information provided is from real-world evidence (RWE), which is an important strength of the study. RWE is increasingly recognized as a vital complement to clinical trial data, particularly in informing the adoption of biosimilars into standard practice.

There have been similar studies before, but this one has two characteristics that make it different. First, it includes a relatively large sample size, with 102 patients, making it one of the largest real-world prospective studies in the neoadjuvant setting, involving dual HER2 blockade with a biosimilar. Second, it is the first time that a biosimilar, trastuzumab CT-P6, is being used in a curative indication in the neoadjuvant setting, which gives the study some additional power at a time when biosimilars are particularly interesting, and establishing a precedent for biosimilar use in early-stage breast cancer.

Taken together, these findings extend beyond clinical outcomes to provide translational evidence supporting the integration of trastuzumab CT-P6 into curative neoadjuvant strategies, highlighting its safety, non-immunogenicity, and potential for biomarker-driven optimization.

## Conclusions

In conclusion, this prospective observational study has shown that trastuzumab CT-P6 is effective, safe and non-immunogenic, and can be used in neoadjuvant treatment in combination with pertuzumab, with pCR data similar to those obtained with the original trastuzumab and without immunogenicity.

## Supplementary Information

Below is the link to the electronic supplementary material.

Supplementary file1 (DOCX 32 KB)

## Data Availability

All anonymized data, along with the scripts used for data analysis, figure generation, and the reproduction of the results presented in this article and its supplementary files, are available on request through the corresponding author and upon approval from the sponsor: Grupo Oncología Clínica y Traslacional–IMIB.

## Abbreviations

AEs: Adverse Events
cN: Clinical node stage
cT: Clinical tumor stage
EMA: European Medicines Agency
ER: Estrogen Receptor
FISH: Fluorescence in situ hybridization
G1: Well differentiated
G2: Moderately differentiated
G3: Poorly differentiated
GI: Gastrointestinal
GPP: Good Pharmacoepidemiology Practices
GX: Not classified
HER2: Human epidermal growth factor receptor
HR: Hormone receptor
IHC: Immunohistochemistry
LVEF: Left ventricular ejection fraction
NACT: Neoadjuvant chemotherapy
NOS: No other specifications
pCR: Pathologic complete response
PR: Progesterone receptor
RCP: Routine clinical practice
RWE: Real World Evidence
Scheme 2: With anthracyclines
Scheme 1 + 3: Without anthracyclines
SLNB: Sentinel lymph node biopsy
ypN: Lymph nodes in axilla
ypT: Residual tumor in breast tissue
