# Supplementary material for: Translational and real-world evidence of trastuzumab biosimilar CT-P6 plus pertuzumab in neoadjuvant HER2-positive early breast cancer
Source: Breast Cancer Res Treat. 2026 Jan 20;215(2):60. doi: 10.1007/s10549-026-07895-8 (PMC12819485; doi:10.1007/s10549-026-07895-8)
Supplement: Supplementary file 1 — Supplementary file1 (DOCX 32 KB) [file 10549_2026_7895_MOESM1_ESM.docx]

**Supplementary Table S1**

**Table S1.** Patient demographics at baseline.

|  | **All patients**  ***n* = 102** | **NACT**  **Scheme 2**  ***n* = 55** | **NACT**  **Scheme 1**  ***n* = 45** | **NACT**  **Scheme 3**  ***n* = 2** |
| --- | --- | --- | --- | --- |
| Age (years), median (range) | 52.00 (30-78) | 56.00 (36-74) | 45.00 (30-72) | 71.50 (65-78) |
| 0 to < 40 | 20 (19.61%) | 6 (10.91%) | 14 (31.11%) | 0 (0.00%) |
| 40 to < 65 | 64 (62.74%) | 37 (67.27%) | 27 (60.00%) | 0 (0.00%) |
| ≥ 65 | 18 (17.65%) | 12 (21.82%) | 4 (8.89%) | 2 (100.00%) |
| Menopausal state |  |  |  |  |
| Pre-menopause | 46 (45.10%) | 17 (30.91%) | 29 (64.44%) | 0 (0.00%) |
| Post-menopause | 56 (54.90%) | 38 (69.09%) | 16 (35.56%) | 2 (100.00%) |
| ER |  |  |  |  |
| Positive | 58 (56.86%) | 33 (60.00%) | 24 (53.33%) | 1 (50.00%) |
| Negative | 44 (43.14%) | 22 (40.00%) | 21 (46.67%) | 1 (50.00%) |
| PR |  |  |  |  |
| Positive | 37 (36.27%) | 23 (41.82%) | 14 (31.11%) | 0 (0.00%) |
| Negative | 65 (63.73%) | 32 (58.18%) | 31 (68.89%) | 2 (100.00%) |
| ER and/or PR |  |  |  |  |
| Positive | 59 (57.84%) | 33 (60.00%) | 25 (55.56%) | 1 (50.00%) |
| Negative | 43 (42.16%) | 22 (40.00%) | 20 (44.44%) | 1 (50.00%) |
| Ki67 |  |  |  |  |
| Ki67<20 | 20 (19.61%) | 11 (20.00%) | 9 (20.00%) | 0 (0.00%) |
| Ki67≥20 | 82 (80.39%) | 44 (80.00%) | 36 (80.00%) | 2 (100.00%) |
| HER2 |  |  |  |  |
| IHC 2+ (FISH amplified) | 20 (19.61%) | 11 (20.00%) | 9 (20.00%) | 0 (0.00%) |
| IHC 3+ | 82 (80.39%) | 44 (80.00%) | 36 (80.00%) | 2 (100.00%) |
| Tumor distribution |  |  |  |  |
| Unique | 68 (66.66%) | 36 (65.44%) | 30 (66.67%) | 2 (100.00%) |
| Multifocal | 19 (18.63%) | 9 (16.36%) | 10 (22.22%) | 0 (0.00%) |
| Multicentric | 14 (13.73%) | 9 (16.36%) | 5 (11.11%) | 0 (0.00%) |
| Unknown | 1 (0.98%) | 1 (1.82%) | 0 (0.00%) | 0 (0.00%) |
| Number of focus |  |  |  |  |
| 1 | 67 (66.34%) | 37 (67.26%) | 28 (63.63%) | 2 (100.00%) |
| 2 | 15 (14.85%) | 7 (12.73%) | 8 (18.18%) | 0 (0.00%) |
| 3 | 6 (5.94%) | 2 (3.64%) | 4 (9.09%) | 0 (0.00%) |
| >4 | 8 (7.84%) | 7 (12.73%) | 1 (2.27%) | 0 (0.00%) |
| Unknown | 5 (4.98%) | 2 (3.64%) | 1 (2.27%) | 0 (0.00%) |
| Size of biggest tumor (mm), median (range) | 30.50 (10-110) | 35.00 (11-90) | 30.00 (10-110) | 43.00 (36-50) |
| 0 to < 20 | 14 (13.72%) | 5 (9.09%) | 9 (20.00%) | 0 (0.00%) |
| 20 to < 50 | 71 (69.61%) | 39 (70.91%) | 31 (68.89%) | 1 (50.00%) |
| ≥ 50 | 17 (16.67%) | 11 (20.00%) | 5 (11.11%) | 1 (50.00%) |
| Histologic grade |  |  |  |  |
| G1 | 9 (8.82%) | 3 (5.45%) | 5 (11.11%) | 1 (50.00%) |
| G2 | 44 (43.14%) | 21 (38.18%) | 22 (48.89%) | 1 (50.00%) |
| G3 | 35 (34.31%) | 29 (52.73%) | 6 (13.33%) | 0 (0.00%) |
| GX | 14 (13.73%) | 2 (3.64%) | 12 (26.67%) | 0 (0.00%) |
| Histological subtype |  |  |  |  |
| Ductal NOS | 94 (92.16%) | 53 (96.36%) | 40 (88.89%) | 1 (50.00%) |
| Other subtypes^a^ | 8 (7.84%) | 2 (3.64%) | 5 (11.11%) | 1 (50.00%) |
| cN |  |  |  |  |
| 0 | 50 (49.02%) | 22 (40.00%) | 27 (60.00%) | 1 (50.00%) |
| 1 | 26 (25.49%) | 16 (29.09%) | 9 (20.00%) | 1 (50.00%) |
| 2 | 16 (15.69%) | 10 (18.18%) | 6 (13.34%) | 0 (0.00%) |
| 3 | 7 (6.86%) | 6 (10.91%) | 1 (2.22%) | 0 (0.00%) |
| x | 3 (2.94%) | 1 (1.82%) | 2 (4.44%) | 0 (0.00%) |
| cT |  |  |  |  |
| 1 | 13 (12.74%) | 7 (12.73%) | 6 (13.33%) | 0 (0.00%) |
| 2 | 66 (64.71%) | 35 (63.63%) | 30 (66.67%) | 1 (50.00%) |
| 3 | 19 (18.63%) | 10 (18.18%) | 8 (17.78%) | 1 (50.00%) |
| 4 | 2 (1.96%) | 1 (1.82%) | 1 (2.22%) | 0 (0.00%) |
| x | 2 (1.96%) | 2 (3.64%) | 0 (0.00%) | 0 (0.00%) |
| Time to surgery (days), median (range) | 68.50 (28-147) | 56.00 (28-140) | 85.50 (47-147) | 76.00 (74-78) |
| Type of surgery^b^ - breast |  |  |  |  |
| Conservative surgery | 63 (62.38%) | 34 (61.82%) | 27 (61.36%) | 2 (100.00%) |
| Mastectomy | 38 (37.62%) | 21 (38.18%) | 17 (36.96%) | 0 (0.00%) |
| Type of surgery^b^ – axilla |  |  |  |  |
| SLNB | 62 (62.63%) | 26 (49.06%) | 34 (77.27%) | 2 (100.00%) |
| Lymphadenectomy | 37 (37.37%) | 27 (50.94%) | 10 (22.73%) | 0 (0.00%) |
| Number of cycles of CT-P6 + pertuzumab, median (range) | -- | 6.0 (4-6) | 4.0 (3-4) | 4.0 (4-4) |

Data are number (%) unless otherwise specified. ^a^ Other histological subtypes include medullar, lobular, apocrine, and invasive carcinoma with micropapillary ductal and papillary type areas. ^b^ Of the 102 patients, 101 underwent breast surgery and 99 underwent axillary surgery. * Indicates statistically significant difference between Scheme 2 and Scheme 1+3 (*p* < 0.05).

cN, clinical node stage; cT, clinical tumor stage; ER, estrogen receptor; FISH, fluorescence *in situ* hybridization; G1, well differentiated; G2, moderately differentiated; G3, poorly differentiated; GX, not classified; HER2, human epidermal growth factor receptor 2; NACT, neoadjuvant chemotherapy; NOS, no other specifications; PR, progesterone receptor; Scheme 2, with anthracyclines; Scheme 1, without anthracyclines; Scheme 3, without anthracyclines; SLNB, sentinel lymph node biopsy
